# A Clinical Decision Support System (KNOWBED) to Integrate Scientific Knowledge at the Bedside: Development and Evaluation Study

**DOI:** 10.2196/13182

**Published:** 2021-03-10

**Authors:** Alicia Martinez-Garcia, Ana Belén Naranjo-Saucedo, Jose Antonio Rivas, Antonio Romero Tabares, Ana Marín Cassinello, Anselmo Andrés-Martín, Francisco José Sánchez Laguna, Roman Villegas, Francisco De Paula Pérez León, Jesús Moreno Conde, Carlos Luis Parra Calderón

**Affiliations:** 1 Group of Research and Innovation in Biomedical Informatics, Biomedical Engineering and Health Economy Institute of Biomedicine of Seville IBiS / Virgen del Rocío University Hospital / CSIC / University of Seville Seville Spain; 2 Publications Department Andalusian Institute of Emergencies and Public Safety Seville Spain; 3 Paediatrics Unit Virgen Macarena University Hospital Seville Spain; 4 Department of Information Systems Coordination Andalusian Health Service Seville Spain; 5 Department of Innovation Technology Virgen del Rocío University Hospital Seville Spain

**Keywords:** evidence-based medicine, clinical decision support system, scientific knowledge integration

## Abstract

**Background:**

The evidence-based medicine (EBM) paradigm requires the development of health care professionals’ skills in the efficient search of evidence in the literature, and in the application of formal rules to evaluate this evidence. Incorporating this methodology into the decision-making routine of clinical practice will improve the patients’ health care, increase patient safety, and optimize resources use.

**Objective:**

The aim of this study is to develop and evaluate a new tool (KNOWBED system) as a clinical decision support system to support scientific knowledge, enabling health care professionals to quickly carry out decision-making processes based on EBM during their routine clinical practice.

**Methods:**

Two components integrate the KNOWBED system: a web-based knowledge station and a mobile app. A use case (bronchiolitis pathology) was selected to validate the KNOWBED system in the context of the Paediatrics Unit of the Virgen Macarena University Hospital (Seville, Spain). The validation was covered in a 3-month pilot using 2 indicators: usability and efficacy.

**Results:**

The KNOWBED system has been designed, developed, and validated to support clinical decision making in mobility based on standards that have been incorporated into the routine clinical practice of health care professionals. Using this tool, health care professionals can consult existing scientific knowledge at the bedside, and access recommendations of clinical protocols established based on EBM. During the pilot project, 15 health care professionals participated and accessed the system for a total of 59 times.

**Conclusions:**

The KNOWBED system is a useful and innovative tool for health care professionals. The usability surveys filled in by the system users highlight that it is easy to access the knowledge base. This paper also sets out some improvements to be made in the future.

## Introduction

Currently, in developed countries, the concept of evidence-based medicine (EBM) is part of medicine itself. In the beginning, the EBM meant a paradigm change in the way that clinical practice was accomplished, leaving a process regarding learning and practice based on static knowledge and authority. However, the EBM concept assumes that the scientific-medical knowledge must emerge from clinical experimentation, and must be used, criticized, and qualitatively interpreted with the best available methodology. Consequently, this knowledge must be essentially dynamic. In the EBM general approach, this knowledge, in conjunction with the clinical experience and the patient’s preferences and data, should directly influence the clinical decision-making process at all the levels of care, considering that the goal of EBM is to improve the patient’s health care quality through enhanced clinical practice [1,2].

Clinical practice is carried out at many complexity levels, so the necessary knowledge to perform it according to the EBM concept must adapt to the real conditions to use the highest quality information possible. The EBM knowledge sources are categorized according to the usability that allows them to be incorporated into the clinical decision-making process at any level in which this process takes place. The usability of the knowledge source shows a direct relationship with the complexity of its methodology, and is therefore better assimilated by the decision process. As a result, the products with detailed information are also more difficult to be incorporated in the health system environment.

Although the EBM supposed a change of attitude in clinical systems, ensuring efficient support to the clinical decisions that must be taken in the patient–doctor relationship context (where it is not easy to consult nor perform an in-depth reading of the original research) was always difficult. In parallel with the EBM conceptual consolidation, some clinical researchers proposed systematic methodologies to achieve products based on the knowledge, to reduce the distance between the research and the practice, thereby saving time for health care professionals in the critical interpretation of the evidence during decision making.

These products were hierarchized in models in 3 proposals. The first one, in 2001, developed a 4-level classification [3]. The second, in 2007, proposed a classification of 5 levels [4]. And the more recent one, in 2009, developed a 6-level classification [5], and has been recently used in relevant research [6,7].

More concretely, the first proposal [3] defined a classification of the following 4 levels:

- Studies: original papers published in journals.
- Syntheses: recompilation of the existing evidence about a specific issue (eg, systematic revisions).
- Synopses: the most relevant elements of a set of evaluated primary studies, including evaluating the methodological quality (eg, the ACP Journal Club).
- Systems: integrate information about the rest of the levels with electronic health records (EHRs).

Comparing this model with the more recent proposal, the 6-level model [5], the main differences between these are (1) the synopses repositories on systematic studies published in scientific reviews that some institutions maintain, and (2) the editorial products that integrate the best practice, in terms of efficacy, according to the explicit and rigorous methods, such as clinical practice guidelines (CPGs) or evidence-based manuals. Probably, CPGs have been the most relevant attempt to inform about the quotidian clinical decisions, and their institutional adoption and individual use are currently accepted criteria for good practice. However, CPGs are complex so their adoption and use are difficult, even in the best of circumstances.

At the top of the pyramid in the 3 proposals, the clinical decision support systems (CDSSs) appear. The CDSSs are the clinical information systems in charge of integrating and summarizing the relevant information about the clinical problems, actualizing, and connecting this information with the patient’s situation. The generalization of the EHR makes possible knowledge integration and records management, allowing the habitual use of the evidence at the patient’s bedside. Incorporating the CDSS in the EHR is a tough challenge that is not solved yet [8,9].

Adopting an EHR by a health care organization involves making organizational decisions to register and maintain patients’ health data, including changes. However, this adoption also makes possible the approach of other types of choices, such as integrating the evidence-based decision support [10].

Consequently, EBM-based interventions improve patient safety. Any clinical intervention must comply with the beneficence principle to the patient, and it is an obligation not to add damage that exceeds the initial clinical condition. Effectiveness and safety are the 2 dimensions that determine the degree of quality of the interventions because no intervention should be assumed to be ineffective even if its cost is zero. The context in which patient care is practiced—the health system—requires improving the effectiveness of interventions and optimizing the efficiency of resources, because health care, whatever its nature, offers a balance between benefits, risks, inconveniences, and costs. Areas such as public health, nursing, and even health policies (called evidence-based health care) have been incorporated into the EBM to ensure the optimal functioning of health systems. It is necessary to extend the dissemination of systematic reviews and clinical guidelines to include electronic access to EHR for all devices, including smartphones [11,12].

EBM contributes to the knowledge in all these dimensions to increase the quality of the intervention. This knowledge is dispersed in many CPGs applied to generalize those actions of proven effectiveness within a specialty or a clinical condition. Despite this, a substantial variation in the provision of services and patient management is documented, between institutions and between professionals of the same institution. The result is known as variability in clinical practice, which can compromise the quality of the services themselves beyond the health care professionals’ actions and the resource allocation equity [13-15]. EBM tends to reduce this variability, promoting the adoption of the most effective, safe, and efficient practices. CDSSs to support translational medicine have been proposed by some researchers [16,17].

The scientific knowledge integration at the bedside with a mobile platform enables health care professionals to make faster and more effective decisions based on validated clinical practice experience. In this sense, a CDSS called the KNOWBED system [18] has been designed, which provides to the health care professional clinically relevant questions concerning the pathology of interest. These questions are associated with recommendations at the bedside, based on the scientific evidence in different existing knowledge bases (eg, massive reference bases, CPGs, systematic reviews). The global architecture of the KNOWBED system is designed as a secure, scalable, standards-based, and EBM service–oriented architecture. Regarding scalability, the infrastructure in which the KNOWBED system has been developed supports more than a dozen similar projects, so it is prepared to receive an even more significant number of users, providing service to all physicians who want to use it within a health system. The fact that the KNOWBED system generates and indexes a set of recommendations from existing scientific evidence, offering intelligent assistance for health professionals, makes it a system based on EBM.

This paper aims to disseminate the KNOWBED project results, highlighting the benefits identified using a CDSS to integrate scientific knowledge at the bedside, encouraging the scientific community to use this kind of system.

The paper is structured as follows: after this introduction, where a brief review of relevant EBM work is presented, we expose the methods carried out. Then, the study results obtained are discussed. Finally, the discussion and conclusions are presented.

## Methods

### Overview of System Components

Functionally, 2 components integrate the KNOWBED system: a web-based knowledge station and a mobile app.

The knowledge station’s actors are the knowledge managers who use the system to manage all the information shown in the mobile app. In other words, the knowledge managers collect the information coming from the existing scientific knowledge in the bibliography and, at the same time, index the clinical recommendations and questions that usually arise throughout the clinical practice, which will be accessible by context based on the HL7 Infobutton standard [19,20]. OpenInfobutton service, from the University of Utah, was used for this task. This service uses contextual information (based on the HL7 Infobutton standard) about the patient, user, clinical setting, and EHR task to anticipate clinicians’ and patients’ information needs. Furthermore, this service retrieves information from online provider reference and patient education resources that may help meet their information needs. This web service exposes an endpoint that receives all the previously detailed information, and returns a JSON format response. This response is processed to offer access through links to the different sources of information provided by it. The effort to deal with the conflict between recommendations from different sources is made by the knowledge manager technologically supported by the knowledge station.

The mobile app allows health care professionals to have access to scientific knowledge from their mobiles device—both smartphones and tablets—as it provides access to questions and clinical recommendations to follow regarding patients’ diagnosis, admission, treatment, etc., indexed by knowledge managers.

### System Technological Architecture

**Figure 1:**
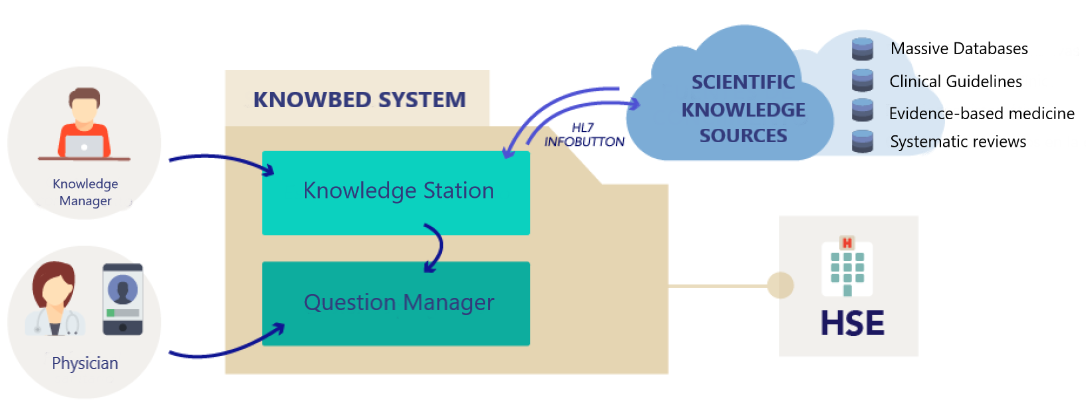
KNOWBED architecture.

From a technological point of view, the KNOWBED system is based on the development and deployment of 2 different modules (Figure 1): the knowledge station and the question manager.

The knowledge station is a web application that allows access to health professionals from the health care centers through their workstations. This web application will be responsible for visualizing, managing, and maintaining the information associated with the knowledge bases defined in the KNOWBED system. For the development of this web application, the Angular 2 framework has been used which, through HTML5, Sass, and TypeScript, allows the development of web applications based on the SPA paradigm (Single-Page-Application). The PostgreSQL relational database engine supported storage and knowledge management, which stores the information associated with questions, recommendations, and suggestions defined for each of the knowledge areas established in the KNOWBED system. The communication between the application and the server uses the HTTP protocol utilizing the Angular 2 built-in HTTP library. The system has communications security based on tokens generated on the server, and managed in the application through the JWT library; these tokens are renewed in each new connection or after a while.

The question manager offers a multiplatform hybrid mobile app. This app has been developed following the IONIC development framework’s premises, capable of developing apps through Angular and Apache Cordova, which provide access to the native mobile phone capabilities. This tool offers professionals the ability to, using their Android mobile devices, access and visualize the set of recommendations defined within the KNOWBED project through a comfortable and intuitive user interface.

For the integration of these different modules, a service-oriented architecture has been implemented. This system focuses on the use of an integration gateway based on the Mirth Connect enterprise service bus. Through this integration gateway, services offering the ability to interoperate remotely with the knowledge have been developed. Several mechanisms have been implemented to access the system from outside of the hospital network and invoke the services published in this integration gateway, based on the corporative LDAP system and the generation of random access tokens. Besides, a set of specific routing rules associated with a reverse proxy working as a gateway to the hospital’s corporate network has been implemented.

Regarding security aspects, the queries to the knowledge bases are based on general parameters such as gender, age, other conditions, the disease, or inpatient/outpatient. The recommendations are generic for this condition, so no personal data of the patient critical to the possibility of identifying the patient are provided. The “Patient data” section was developed as a link to the EHR application, and this can be used only when connected to the secure corporate network.

### Selected Use Case

A specific use case was selected to validate the KNOWBED system: the bronchiolitis pathology from the Paediatrics Unit of the Virgen Macarena University Hospital (Seville).

Bronchiolitis is a common viral infection of the lower respiratory tract that affects children under 2 years of age. This pathology is characterized by acute infection and inflammation of the small airways in the lungs [21,22]. It is the most frequent cause of non-elective admission to the intensive care unit [23,24]. Other researchers have performed studies to improve bronchiolitis management using the technology [25,26].

Based on these considerations, and considering this pathology has a greater incidence during the winter months [27], the pilot was carried out between December and February. In this way, the system was more frequently used and more useful for health care professionals’ clinical decision making.

### System Evaluation

The system was evaluated using 2 indicators: usability and efficacy.

The KNOWBED system usability was assessed to evaluate the health care professionals’ acceptance, using an ad hoc survey asking users regarding the functionalities (Multimedia Appendix 1). The survey recorded sociodemographic information (sex, date of birth, and job title) as well as 13 items that were answered with a 10-point Likert scale (1=strongly disagree; 10=strongly agree) [28]. The survey was administered in 2 phases: phase 1, before using the technological system to know their expectations of the system before interacting with it; and phase 2, to learn about their experience after using the mobile app. Likewise, when new health care professionals joined the Paediatrics Unit, they were informed about the mobile app and were invited to use it.

By contrast, the system’s efficacy was studied by analyzing the percentage of acceptance of the recommendations generated by the system. This acceptance was studied by launching the following question when leaving the system: “You are going to leave the App, was this App useful to you?”

## Results

### Development and Evaluation of the KNOWBED System

The KNOWBED system has been developed to incorporate scientific evidence into daily clinical practice, improving patient care and providing health care professionals with recommendations based on up-to-date and relevant scientific knowledge.

**Figure 2:**
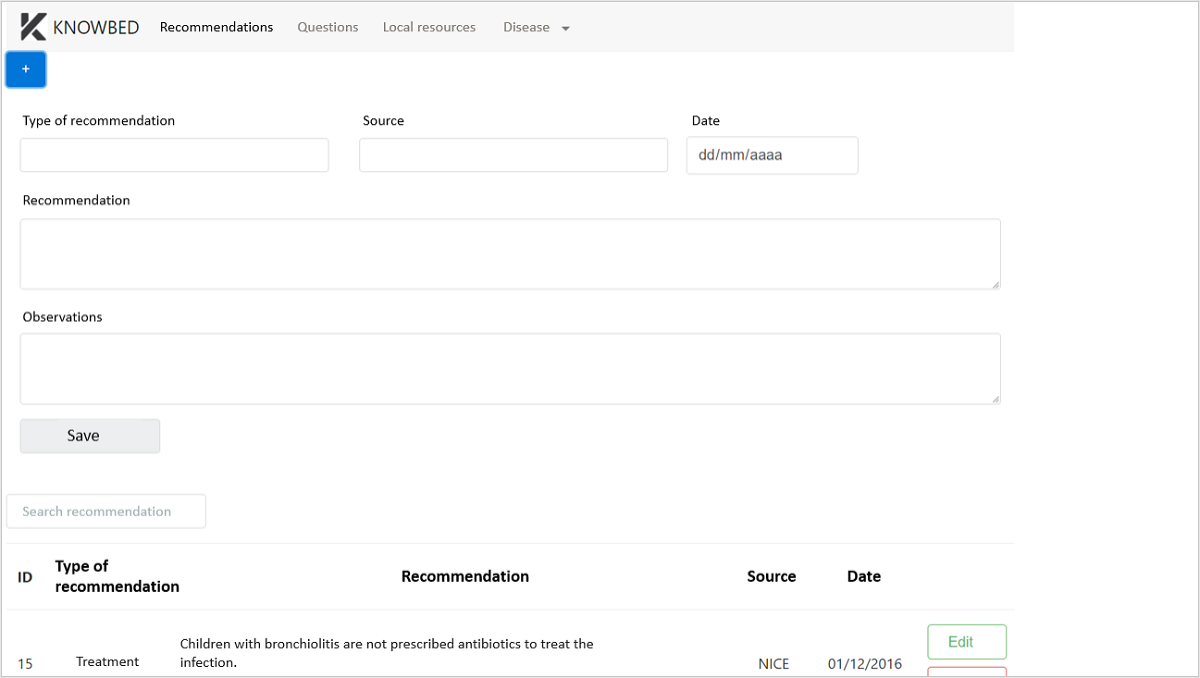
KNOWBED knowledge station.

A screenshot of the KNOWBED knowledge station is shown in Figure 2, which presents the section to add new recommendations, specifying the type, the source, the date, and possible observations.

**Figure 3:**
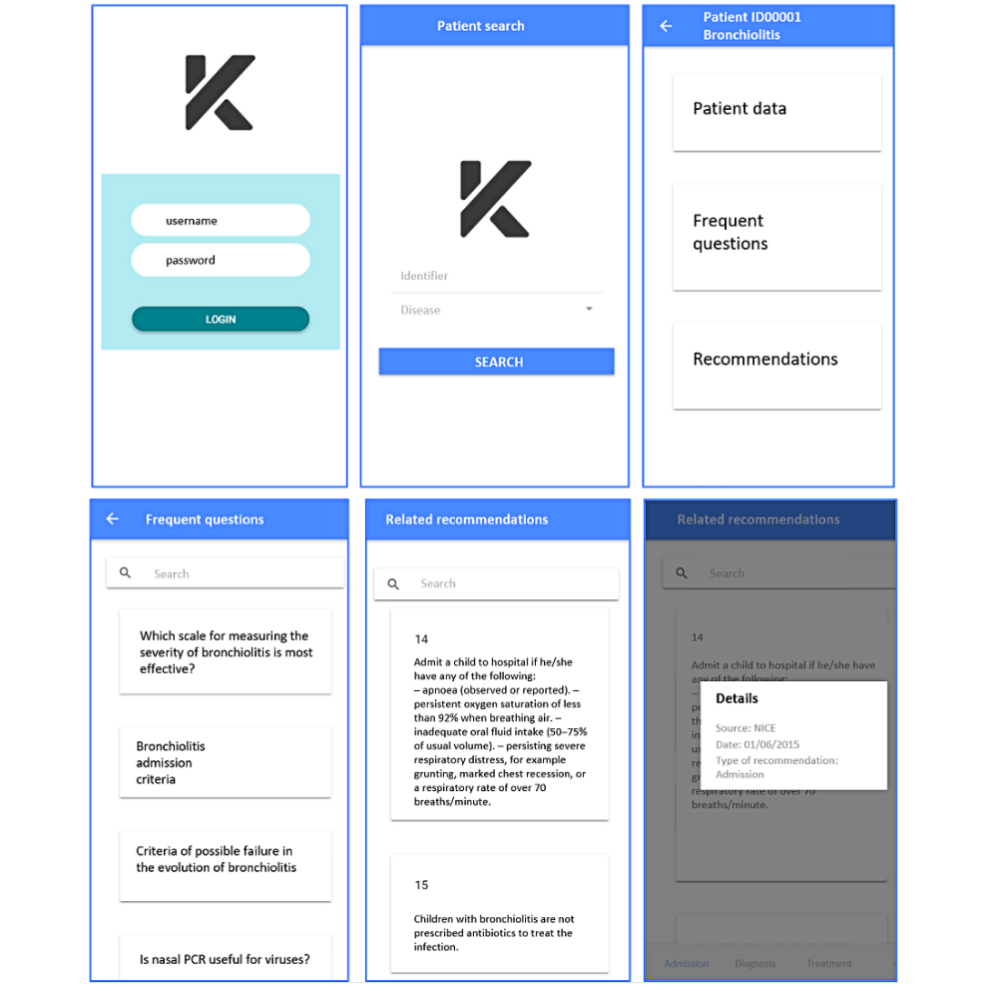
KNOWBED mobile app.

Some screenshots of the KNOWBED mobile app are shown in Figure 3: On the upper left side, the login section is shown. The patient search is displayed in the upper middle section. In the upper right section, the main menu regarding a specific patient is shown. The list of frequent questions regarding this pathology created by the knowledge manager is shown in the bottom left. In the bottom middle, the list of recommendations related to a specific question is displayed. The details of a particular recommendation, including the source, the date, and the type, are shown in the bottom right.

It is also relevant to mention that the KNOWBED system can be integrated for its exploitation in other health care centers.

Furthermore, a methodology to incorporate a new pathology into the knowledge station has been defined. In this sense, a knowledge manager can include further information, and new questions and recommendations could support a health care professional regarding other pathologies.

### System Evaluation

To assess the system usage, the number of times users have used the mobile app was analyzed. During the 3-month pilot, the results show that 15 health care professionals made use of it, having registered up to 59 accesses, 23 of which took place after the pilot period.

**Figure 4:**
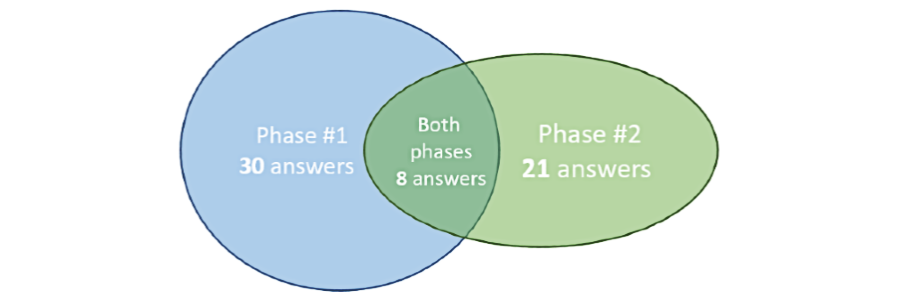
Usability survey: Groups and quantities of responses.

Regarding the usability survey (Multimedia Appendix 1), in phase 1, 30 health care professionals answered the survey, but of them, only 8 completed it in phase 2. However, as mentioned in the “System Evaluation” section, new health care professionals joined the Paediatrics Unit during the pilot, and they used the system. More specifically, 13 health care professionals were incorporated, and they answered the survey after using the system (ie, only in phase 2). In this way, 8 surveys were filled-in for both phase 1 and phase 2, while 13 surveys were filled-in only for phase 2. Figure 4 shows the groups and numbers of health care professionals who responded to phases 1 and 2.

Additionally, 5 health care professionals interested in using the system could not use it because they had iOS phones.

In those users where a comparative analysis can be done (ie, in cases where they have answered in both phases), the results show the following:

- In 7 of 13 questions, the expectations were somewhat higher.
- In 2 of the 13 questions, the expectations were lower.
- In 3 of the 13 questions, the expectations coincided with what was experienced after using the system.

On the one hand, for the health care professionals who have answered in the second phase exclusively (n=13), a comparison of the expectation before and after using the system was not possible. Upon reviewing their opinion after using the system, it is relevant to highlight that the best-scored questions related to the organization support (item number 13; score 8.63/10), and to the improvement in the time spent for decision making (item number 10; score 8.46/10):

- (item number 13) “Overall, I think the organization where I work would support the use of the KNOWBED App.”
- (item number 10) “The KNOWBED App can help me resolve some clinical decisions quickly.”

On the other hand, the worst-scored question (item number 9; score 7.46/10) was: “I think I will have the technical assistance available to solve problems associated with the KNOWBED App.”

The system’s efficacy has not been revealed because none of the users have answered the question when leaving the mobile app, so no data on efficacy are available.

## Discussion

The study’s main findings are the design, development, deployment, and validation of a CDSS called the KNOWBED system to integrate scientific knowledge at the bedside. This system can be presented as an innovative and useful tool due to clinical decision making being offered, allowing health care professionals to access recommendations based on scientific evidence at the bedside by using a mobile device.

A limitation of this study is that the number of answered usability surveys has been small. However, 23 of the accesses that health care professionals made (out of 59 total accesses) have taken place after the pilot period. Consequently, the affirmation of the “KNOWBED system is useful even in months of a lower incidence of this pathology” has been concluded.

This experience with the KNOWBED system concludes that if pathologies with more incidence than bronchiolitis are included, the technological system will be useful for clinical decision making. Furthermore, bronchiolitis is a pathology whose clinical protocols are very well defined, so consulting the literature based on evidence is perhaps less relevant than other pathologies for which clinical protocols are less defined. This fact explains why the 15 users have only registered 59 accesses to the mobile app.

As future work, to continue analyzing the system’s usability, encouraging health care professionals’ consciousness-raising about the importance of answering the usability survey is relevant, both in the preuse phase of the technology and in the postuse phase, to obtain important data on the usability of technologies.

Furthermore, as future work, it should be stressed that it is required to answer the final question about the usefulness of the mobile app. This indicator was not utilized in this first pilot because the health care professionals have not answered the final question.

The next stage will be extending the experience to more health care centers and including other pathologies, making it possible to increase the number of health care professionals for whom the KNOWBED system’s use may be useful and relevant.

The pilot has highlighted a technological-level limitation: the KNOWBED system should have been developed for the iOS operating system as well. During the pilot execution, 5 of the potential users interested in using the mobile app could not make use of it as it was not available for Apple devices.

As an improvement to the knowledge station, the acceptance of all knowledge managers of a specific pathology will be required to validate any information inclusion/modification in the system, and this validation must be done before that new information is reflected in the mobile app. Moreover, nonfree bibliographic bases will be included to improve the knowledge base by feeding their information as well.

Currently, HL7 International is working on an HL7 project called The Fast Healthcare Interoperability Resources (FHIR) for EBM Knowledge Assets project (EBMonFHIR), sponsored by the HL7 Clinical Decision Support Work Group and co-sponsored by the HL7 Clinical Quality Information Work Group and Biomedical Research and Regulation Work Group. The goal of EBMonFHIR is to provide interoperability for those producing, analyzing, synthesizing, disseminating, and implementing evidence of clinical research and recommendations for clinical care included in the CPGs. EBMonFHIR could be a new relevant standard to take into account in the KNOWBED system.

## Appendix

Multimedia Appendix 1 Usability survey.

## Abbreviations

CDSS: clinical decision support system
CPG: clinical practice guideline
EBM: evidence-based medicine
EBMonFHIR: FHIR for EBM Knowledge Assets project
EHR: electronic health record
FHIR: Fast Healthcare Interoperability Resources
