# Supplementary material for: A Clinical Decision Support System (KNOWBED) to Integrate Scientific Knowledge at the Bedside: Development and Evaluation Study
Source: JMIR Med Inform. 2021 Mar 10;9(3):e13182. doi: 10.2196/13182 (PMC7991993; doi:10.2196/13182)
Supplement: Multimedia Appendix 1 [file medinform_v9i3e13182_app1.pdf]

Proyecto KNOWBED

Cuestionario de usabilidad de la App KNOWBED

Estimado/a compañero/a:

En el marco del proyecto KNOWBED, hemos desarrollado un cuestionario para evaluar la usabilidad percibida por los/as profesionales sanitarios/as a la hora de adoptar la App KNOWBED desarrollada.

Este cuestionario es anónimo. La primera parte del cuestionario se refiere sus características sociodemográficas, y la segunda parte está formada por los ítems del mismo.

Todos los ítems son obligatorios.

Gracias por su colaboración.

Sexo: □ ***Mujer*** □ ***Hombre***

Fecha de nacimiento: ___/___/______

Puesto de trabajo: □ ***MIR*** □ ***Adjunto o FEA*** □ ***Otros:*** ________________

A continuación valore de 1a 10 cada pregunta, puntuando 1 si está totalmente en desacuerdo y 10 si está totalmente de acuerdo. Estas preguntas están relacionadas en la atención de pacientes con Bronquiolitis.

1. Tengo la intención de consultar la opción 'preguntas frecuentes' en la App KNOWBED cuando sea necesario

1 (totalmente en desacuerdo)	*2*	*3*	*4*	*5 (neutro)*	*6*	*7*	*8*	*9*	*10 (totalmente de acuerdo)*

2. Tengo la intención de consultar la opción 'recomendaciones' sobre bronquiolitis en la App KNOWBED cuando sea necesario

1 (totalmente en desacuerdo)	*2*	*3*	*4*	*5 (neutro)*	*6*	*7*	*8*	*9*	*10 (totalmente de acuerdo)*

3. Tengo la intención de realizar búsquedas en bases de datos bibliográficas (PubMed, MedLine+) a través de la App KNOWBED

1 (totalmente en desacuerdo)	*2*	*3*	*4*	*5 (neutro)*	*6*	*7*	*8*	*9*	*10 (totalmente de acuerdo)*

4. La App KNOWBED me permitiría utilizar más ágilmente las Apps PediaMecum, PediAPP, CIMA, Scores Pediatría

1 (totalmente en desacuerdo)	*2*	*3*	*4*	*5 (neutro)*	*6*	*7*	*8*	*9*	*10 (totalmente de acuerdo)*

5. Si necesito sugerir cambios en las preguntas, recomendaciones o recursos ofrecidos, tengo la intención de notificarlo a través de la App KNOWBED

1 (totalmente en desacuerdo)	*2*	*3*	*4*	*5 (neutro)*	*6*	*7*	*8*	*9*	*10 (totalmente de acuerdo)*

6. Creo que la información presentada en la App KNOWBED es clara

1 (totalmente en desacuerdo)	*2*	*3*	*4*	*5 (neutro)*	*6*	*7*	*8*	*9*	*10 (totalmente de acuerdo)*

7. La App KNOWBED podría mejorar mi rendimiento en el trabajo

1 (totalmente en desacuerdo)	*2*	*3*	*4*	*5 (neutro)*	*6*	*7*	*8*	*9*	*10 (totalmente de acuerdo)*

8. En general, creo que la App KNOWBED es fácil de usar

1 (totalmente en desacuerdo)	*2*	*3*	*4*	*5 (neutro)*	*6*	*7*	*8*	*9*	*10 (totalmente de acuerdo)*

9. Creo que tendré la asistencia técnica disponible para solucionar problemas asociados a la App KNOWBED

1 (totalmente en desacuerdo)	*2*	*3*	*4*	*5 (neutro)*	*6*	*7*	*8*	*9*	*10 (totalmente de acuerdo)*

10. La App KNOWBED me puede ayudar a resolver con mayor rapidez algunas decisiones clínicas

1 (totalmente en desacuerdo)	*2*	*3*	*4*	*5 (neutro)*	*6*	*7*	*8*	*9*	*10 (totalmente de acuerdo)*

11. La App KNOWBED puede mejorar la asistencia sanitaria de mis pacientes

1 (totalmente en desacuerdo)	*2*	*3*	*4*	*5 (neutro)*	*6*	*7*	*8*	*9*	*10 (totalmente de acuerdo)*

12. Tengo la intención de utilizar la App KNOWBED cuando esté disponible en mi centro

1 (totalmente en desacuerdo)	*2*	*3*	*4*	*5 (neutro)*	*6*	*7*	*8*	*9*	*10 (totalmente de acuerdo)*

13. En general, creo que mi centro apoyaría el uso de la App KNOWBED

1 (totalmente en desacuerdo)	*2*	*3*	*4*	*5 (neutro)*	*6*	*7*	*8*	*9*	*10 (totalmente de acuerdo)*
